# Cross-Sectional and Longitudinal Validity of Automated Body Composition Methods in U.S. Air Force Reserve Officers’ Training Corps Cadets Throughout a 12-Week Physical Training Program: The Supporting Tactical Athlete Readiness Program

**DOI:** 10.1093/milmed/usag107

**Published:** 2026-04-03

**Authors:** Austin J Graybeal, Sheena Puleali’i, Christian Rodriguez, Antonella V Schwarz, Molly F Johnson, Maria G Kaylor, Andrew Addison, Hunter Haynes

**Affiliations:** Department of Kinesiology, Harris College of Nursing and Health Sciences, Texas Christian University, Fort Worth, TX 76129, United States; Department of Military Science & Leadership, College of Arts and Sciences, University of Southern Mississippi, Hattiesburg, MS 39406, United States; Pennington Biomedical Research Center, LSU System, Baton Rouge, LA 70808, United States; Department of Kinesiology & Sports Sciences, College of Health Professions & Medical Sciences, Barry University, Miami Shores, FL 33161, United States; School of Kinesiology & Nutrition, College of Education and Human Sciences, University of Southern Mississippi, Hattiesburg, MS 39406, United States; School of Kinesiology & Nutrition, College of Education and Human Sciences, University of Southern Mississippi, Hattiesburg, MS 39406, United States; School of Kinesiology & Nutrition, College of Education and Human Sciences, University of Southern Mississippi, Hattiesburg, MS 39406, United States; School of Kinesiology & Nutrition, College of Education and Human Sciences, University of Southern Mississippi, Hattiesburg, MS 39406, United States

## Abstract

**Introduction:**

Accurate assessment of body composition is essential for evaluating and maintaining military readiness among U.S. Air Force Reserve Officers’ Training Corps (AFROTC) cadets, who must meet the same standards as active-duty personnel. Current reliance on BMI and circumference-based methods often leads to misclassification because of measurement variability and the inability to distinguish between lean and fat mass. Although laboratory methods like dual-energy X-ray absorptiometry (DXA) are ideal, their high cost, limited portability, and the need for trained personnel make them impractical for field use. During training or deployment, these limitations also hinder consistent monitoring when access to fitness and nutrition resources is most restricted. Therefore, this study examined the cross-sectional and longitudinal accuracy of body fat percentage (BF%) estimates obtained from the following emerging, field-based technologies compared with DXA and current AFROTC circumference-based methods in AFROTC cadets assessed before and after a 12-week training program: bioelectrical impedance spectroscopy (BIS), near-infrared spectroscopy (NIRS), smartwatch bioelectrical impedance analysis (SWBIA), and smartphone-based three-dimensional optical scanning (3DO).

**Materials and Methods:**

At baseline before the 12-week training program, 57 cadets (26 F, 31 M; BF%: 27.2 ± 9.5%) underwent body composition assessments using DXA, circumference-based equations, BIS, NIRS, SWBIA, and 3DO to assess cross-sectional validity. 3DO-derived circumferences were also substituted for manual measures in circumference-based equations. A subset of 50 cadets returned after the program to assess longitudinal validity, defined as the agreement between the change in the BF% from DXA and those produced by each field method. Cross-sectional and longitudinal validity were assessed using null-hypothesis significance and equivalence testing, coefficients of determination, concordance correlation coefficients, root mean square error, Bland-Altman analyses, and Deming regression. Classification accuracy based on AFROTC BF% standards was assessed at baseline using sensitivity and specificity analyses. The University Institutional Review Board approved the study, and informed consent was obtained before participation.

**Results:**

Although most cadets met the AFROTC BMI standards (57.9%), only one-third met the DXA-derived BF% standards (33.3%), indicating false compliance when using BMI alone. Cross-sectionally, NIRS and SWBIA demonstrated the lowest measurement error and the highest accuracy for classifying cadets according the AFROTC BF% guidelines. Longitudinally, changes in BF% from 3DO and NIRS demonstrated the strongest agreement with DXA. Overall performance comparisons indicated that NIRS, SWBIA, and 3DO provided the most accurate and consistent BF% estimates relative to DXA, while traditional circumference-based equations produced the largest errors.

**Conclusions:**

NIRS, SWBIA, and 3DO provide accurate, portable alternatives to DXA for assessing BF% in AFROTC cadets. Despite most cadets meeting BMI standards and the widespread use of circumference-based equations in practice, our findings that the majority of cadets exceeded DXA-derived BF% standards, and that circumference-based estimates were the least accurate, highlight the limitations of current assessment methods. Strengths include direct comparison with DXA and both cross-sectional and longitudinal assessments, although limitations include reliance on a single cohort and modest BF% changes. Overall, these findings support integrating these accessible, user-friendly technologies into military health and fitness assessments, with future studies warranted in larger cohorts undergoing differential training programs across varied settings.

## INTRODUCTION

Reserve Officers’ Training Corps (ROTC) programs are designed to mentally and physically prepare cadets for the demands of a military career.^1^ An important element of this preparation is cultivating a culture of physical capability through structured, time-intensive physical training (PT) programs. These programs aim to develop deployment-ready cadets by improving their physical fitness, instilling discipline, unity, and promoting body composition changes (lower body fat percentage [BF%], greater lean mass) that align with commissioning standards and the demands of military service.^1^ Body composition serves as a key indicator of overall health, physical readiness, and the ability to carry out necessary tasks required by the military.^2^ As a result, body composition standards for BMI and BF% estimates are mandated to motivate fitness behaviors, prevent obesity, and ensure a physically ready force.^3^ Consequently, service members are now required to maintain body composition standards throughout their military careers, often beginning in ROTC, with failure potentially resulting in administrative action ranging from performance improvement plans to separation from service.^2^^,^^4^

Despite established standards, rising military obesity rates remain a concern.^5^^,^^6^ Beyond the well-known health risks of obesity, increased adiposity is also associated with greater injury risk, posing another obstacle to recruiting and retaining military personnel.^5^^,^^7^^,^^8^ As such, accurately monitoring body composition is an essential component for assessing and establishing physical readiness.^5^ However, the limitations of accurate assessment methods are evident in the reliance on BMI, which often introduces measurement biases when classifying military personnel as overweight or obese.^5^ Yet, because of its ease of implementation, most military branches still rely on BMI to establish these standards.^5^ Thus, is it unsurprising that the U.S. Armed Forces continues to experience substantial challenges recruiting individuals that meet the fitness standards required for eligibility, particularly among young adults who represent the majority of military applicants.

In most military training programs, body composition also serves as a qualification criterion for enlistment, load carriage, and duty fulfillment,^2^^,^^9^ where cadets must meet the same standards as active-duty personnel. Given that the structured PT mandated by most ROTC programs is designed to increase skeletal muscle mass, relying solely on BMI to assess cadets may overestimate the incidence of overweight and obesity among cadets with greater muscularity,^5^ potentially disqualifying otherwise deployment-ready individuals or discouraging cadets from building muscle due to fear of dismissal. Conversely, BMI may underestimate risk in cadets with low body weight and insufficient skeletal muscle, potentially granting underprepared cadets’ eligibility. This misclassification could result in inefficient use of defense resources and elevate the risk of adverse outcomes in mission-critical situations. Therefore, it is critical to evaluate and identify valid body composition methods to more effectively monitor these important metrics in military training programs.

Although validated laboratory methods such as dual-energy X-ray absorptiometry (DXA) are ideal, they are impractical for ROTC populations because of cost, accessibility, and the need for trained personnel. Moreover, these methods face geographical constraints that limit their portability; not only during deployments when body composition fluctuates because of restricted access to training and nutrition resources, but also at home stations, which can span any of the six continents hosting U.S. military bases. For these reasons, manual circumference measurements are commonly used to estimate BF% in cadets and active-duty service members,^5^ as their simplicity enables rapid assessment of large units without specialized equipment and regardless of location. However, manual circumference assessments inherently suffer from limited reliability because of susceptibility to human error, lack of standardization, and variability among personnel. In military populations specifically, frequent deployments and relocations often lead to high staff turnover, which can undermine measurement quality and interrater reliability when multiple personnel conduct manual assessments across cohorts and over time. Therefore, more sophisticated yet convenient non-invasive field methods such as handheld near-infrared spectroscopy (NIRS), smartwatch bioelectrical impedance analysis (SWBIA), and smartphone-derived three-dimensional optical imaging (3DO) are appealing because of cost, accessibly, and speed of administration when compared to laboratory techniques.^10^ Therefore, if these devices agree with those derived from more sophisticated methods such as DXA, they could serve as viable tools for assessing body composition in military personnel, offering greater accuracy and consistency when used across geographical locations and among multiple assessors.^5^

Although evaluating the cross-sectional agreement between body composition techniques holds some utility, longitudinal investigations are needed to establish the relative comparability of specific methods for quantifying changes in distinct body compartments over time.^11^^,^^12^ For example, a given body composition method could demonstrate suitable cross-sectional validity (relative to a ­criterion method) yet be unable to sufficiently detect longitudinal body composition changes. Aside from determining eligibility for ROTC cadets, changes in body composition, particularly in response to exercise or nutrition interventions, are likely of greater interest to practitioners than the validity of a single estimate at a specific timepoint.^12^ Establishing the longitudinal validity of these techniques would allow for further, more accurate evaluations of both body composition and physical readiness, ultimately supporting the cadre in designing targeted training programs and effectively monitoring progress.^13^

Currently, most body composition research in tactical populations focuses on cross-sectional validity and primarily involves active-duty Army personnel, highlighting the need for longitudinal evaluations in other military branches and training programs such as ROTC.^2-4^^,^^9^^,^^10^^,^^14^ To our knowledge, no studies have evaluated the validity of both research- and consumer-grade body composition methods against a criterion model in U.S. Air Force ROTC (AFROTC) cadets undergoing a longitudinal PT program. As such, the purpose of this study was to determine the cross-sectional and longitudinal validity of contemporary body composition estimation techniques in a group of AFROTC cadets over the course of a 12-week resistance training and conditioning program.

## METHODS

This project examined the agreement between body composition measurement techniques collected throughout the STAR Program, a strength and conditioning intervention implemented within an AFROTC detachment. The present analysis represents an independent component of the broader research initiative that is methodologically and analytically distinct from the parent project. Specifically, the primary outcomes assessed here—agreement amongst consumer- and research-grade body composition estimation techniques—are unique to this investigation and do not overlap with the primary endpoints of the larger study. Only a limited set of secondary variables, such as participant demographics and program characteristics, are shared across iterations of this study. The study procedures were conducted according to the guidelines laid down in the Declaration of Helsinki, and all procedures involving human subjects were approved by the University Institutional Review Board (IRB#24-0476). Written informed consent was obtained from all cadets before participation.

### Cadets

Given that sample sizes in studies like the one presented here are constrained by AFROTC enrollment and semester-specific attrition, our final sample size was determined by the number of cadets available within the detachment during the study period. However, using a moderate effect size (*d* = 0.50) for a two-tailed, paired samples *t*-test, a priori sample size calculations revealed that *n* = 34 would yield 80.7% power when comparing cross-sectional BF% estimates between methods; the study’s primary outcome.^15^ Seventy-seven cadets enrolled in the AFROTC program were prospectively recruited for participation. Eligibility criteria included being ≥18yrs, medically cleared for PT, and actively enrolled in the AFROTC program. Cadets were excluded if they were <18yrs; lacking medical clearance for PT; not enrolled in the AFROTC program; pregnant; planning to become pregnant; breastfeeding/lactating; having a pacemaker/other electrical implant; any neuromuscular or musculoskeletal injury, impairment, or surgery that would preclude participation in training; or missing any limb or part of a limb.

Among the 63 cadets who completed all baseline body composition assessments, two were excluded because of device errors preventing comparisons for ≥1 device, and four were excluded as outliers (±3SD for ≥1 device).^16^ Of the remaining 57 cadets, seven withdrew before follow-up testing for personal reasons. Ultimately, 57 cadets completed baseline testing and were included in the cross-sectional analysis, whereas 50 cadets completed both baseline and follow-up testing for longitudinal analysis. Cadet characteristics, as well as BF% estimates at baseline and the change in BF% (ΔBF%) at follow-up, are presented in Table 1.

**Table 1.** Cadet Characteristics and the Cross-Sectional and Longitudinal Validity of Each Body Composition Assessment Method Relative to DXA

Cross-sectional validity (*N* = 57)	Mean (95% CI)	**Met cutoff—lower *N* (%)** ^a^	**Met cutoff—upper *N* (%)** ^a^	**CE (95% CI)** ^b^	R^2^	CCC	RMSE	SEE	**EQ (90% CI)** ^c^
Age (y)	20 (19, 21)								
Height (cm)	171.4 (168.5, 174.2)								
Weight (kg)	73.1 (68.7, 77.5)								
BMI (kg/m^2^)	24.7 (23.6, 25.9)	33 (57.9%)	42 (73.7%)						
DXA BF% (Criterion)	27.2 (24.7, 29.8)	19 (33.3%)	36 (63.2%)						
BIS BF%	26.8 (24.8, 28.9)	15 (26.3%)	41 (71.9%)	−0.39 (−1.78, 1.01)	0.70	0.81	5.22	2.89	Y (−1.55, 0.78)
AFROTC_STANDARD_ BF%	21.3 (18.3, 24.2)	30 (52.6%)	48 (84.2%)	−5.96 (−7.33, −4.60)*	0.78	0.75	7.84	3.64	N (−7.10, −4.82)
AFROTC_3DO_ BF%	25.4 (22.6, 28.2)	20 (35.1%)	40 (70.2%)	−1.85 (−3.36, −0.33)*	0.71	0.82	5.96	3.23	N (−3.11, −0.58)
Smartphone 3DO BF%	23.8 (21.8, 25.8)	22 (38.6%)	50 (87.7%)	−3.45 (−4.68, −2.21)*	0.76	0.79	5.77	2.82	N (−4.48, −2.41)
Smartwatch BIA BF%	27.2 (25.0, 29.4)	14 (24.6%)	41 (71.9%)	−0.02 (−1.12, 1.09)	0.81	0.89	4.13	1.81	Y (−0.94, 0.91)
NIRS BF%	23.3 (21.2, 25.4)	25 (43.9%)	49 (86.0%)	−3.95 (−4.74, −3.17)*	0.91	0.86	4.93	1.44	N (−4.61, −3.30)

Longitudinal validity (*N* = 50)	Mean (95% CI)	Met cutoff—lower *N* (%)^a^	Met cutoff—upper *N* (%)^a^	CE (95% CI)^b^	R^2^	CCC	RMSE	SEE	EQ (90% CI)^c^
Age (y)	20 (19, 21)								
Height (cm)	172.1 (169.1, 175.1)								
Weight (kg)	72.9 (68.0, 77.7)								
BMI (kg/m^2^)	24.6 (23.5, 25.8)	29 (58.0%)	39 (78.0%)						
ΔDXA BF% (Criterion)	−0.85 (−1.38, −0.32)	17 (33.3%)	38 (74.5%)						
ΔBIS BF%	−0.21 (−1.36, 0.95)	12 (24.0%)	37 (74.0%)	0.64 (−0.51, 1.79)	0.06	0.18	4.04	3.92	N (−0.32, 1.60)
ΔAFROTC_STANDARD_ BF%	−0.60 (−1.42, 0.22)	31 (62.0%)	45 (90.0%)	0.25 (−0.47, 0.97)	0.25	0.45	2.53	2.19	Y (−0.35, 0.85)
ΔAFROTC_3DO_ BF%	0.10 (−0.57, 0.76)	18 (36.0%)	40 (80.0%)	0.95 (0.34, 1.55)*	0.27	0.46	2.30	1.97	N (0.44, 1.45)
ΔSmartphone 3DO BF%	−0.01 (−0.57, 0.55)	22 (44.0%)	46 (92.0%)	0.84 (0.29, 1.39)*	0.25	0.45	2.09	1.82	N (0.38, 1.30)
ΔSmartwatch BIA BF%	−0.38 (−1.24, 0.47)	12 (24.0%)	38 (76.0%)	0.47 (−0.32, 1.25)	0.18	0.38	2.78	2.51	N (−0.19, 1.12)
ΔNIRS BF%	0.01 (−0.52, 0.55)	21 (42.0%)	44 (88.0%)	0.86 (0.29, 1.43)*	0.18	0.38	2.17	1.97	N (0.39, 1.34)

Data are presented as mean (95% CI).

Abbreviations: 3DO, smartphone-based three-dimensional optical scanning; AFROTC, U.S. Air Force Reserve Officer Training Corps; AFROTC_3DO_, AFROTC circumferences-based method with 3DO circumferences substituted for manual measurements; AFROTC_STANDARD_, AFROTC circumferences-based method using manual circumference measurements; BF%, body fat percentage; BIA, bioelectrical impedance analysis; BIS, bioelectrical impedance analysis; BMI, body mass index; CCC, concordance correlation coefficient; CE, constant error; CI, confidence internal; DXA, dual-energy X-ray absorptiometry; EQ, equivalence; NIRS, near-infrared reactance spectroscopy; RMSE, root mean square error; SEE, standard error of the estimate; Δ, change pre and post training program.

a Lower cutoff defined as BMI < 25 kg/m^2^, or BF% < 18% for males and < 26% for females; upper cutoff defined as BMI < 27 kg/m^2^, or BF% < 26% for males and < 36% for females.

b Calculated as the method in question minus the criterion (DXA).

c Cross-sectional equivalence bounds: ±2%; longitudinal equivalence bounds: ±1%.

* Significantly different from DXA at *P* < .050.

Of the 57 cadets included the cross-sectional analyses, 54.4% were male (*n* = 31), and 42.1% (*n* = 24), 19.3% (*n* = 11), 31.6% (*n* = 18), and 7.0% (*n* = 4) were freshman, sophomores, junior, and seniors, respectively. Additionally, 52.6% (*n* = 30), 38.6% (*n* = 22), 7.0% (*n* = 4), and 1.8% (*n* = 1) were Black, White, Hispanic, and Asian, respectively. Of the 50 cadets that returned at follow-up, 56% (*n* = 28) were male, and 40% (*n* = 20), 22% (*n* = 11), 32% (*n *= 16), and 6% (*n* = 3) were freshman, sophomores, juniors, and seniors. Moreover, 50% (*n* = 25), 42% (*n* = 21), 6% (*n* = 3), and 2% (*n* = 1) were Black, White, Hispanic, and Asian.

### Procedures

BF% was assessed at two time points: baseline before program initiation and following the 12-week PT program. At baseline, cadets reported to the laboratory between 0500-1100 after an ≥8-hour overnight fast from food and beverage and a ≥ 12-hour abstention from exercise. Upon arrival, cadets wore tight-fitting athletic clothing and removed shoes, accessories, and all external metal. Then, measurements of height and weight were collected using a digital scale and stadiometer (seca 286, SECA, Hamburg, Germany), neck (NC), waist (WC), and hip circumferences (HC) using tape measure, and body composition estimates using DXA, bioelectrical impedance spectroscopy (BIS), SWBIA, 3DO, and NIRS. Following the 12-week training program, remaining cadets completed follow-up testing using identical procedures. To ensure consistency, follow-up assessments were scheduled within ±1-hour of each participant’s baseline testing time.

After collecting all anthropometric and body composition estimates at each timepoint, cadets were classified as either meeting or not meeting the U.S. Air Force (USAF) body composition standards. BMI cutoffs ranged from no more conservative than <25kg/m^2^ to no more liberal than <27kg/m^2^, and BF% standards ranged from <18% to <26% for males and <26% to <36% for females.^17^

### Dual-Energy X-Ray Absorptiometry

BF% estimates derived from DXA (Lunar iDXA, General Electric, Boston, MA, United States) were used as the criterion BF% measurement. DXA assessments were conducted in accordance with established guidelines,^18^ and reflection scanning procedures were used for larger cadets.^19^^,^^20^

### Bioelectrical Impedance Spectroscopy

A tetrapolar hand-to-foot BIS device (SFB7, Impedimed, Carlsbad, CA, United States) was used to measure BF% using established guidelines.^21^^,^^22^ After ≥7-minutes in the supine position, cadets were instructed to lay with arms and legs spread away from the torse while adhesive electrodes were placed on the cadets’ left hand and foot. Device coefficients (sex, body density/proportion, hydration) were those recommended by the manufacturer,^23^ and Cole plots were visually inspected after each test to ensure measurement quality.

### Smartphone 3DO Scanning Application

BF%, as well as select circumferences relevant to the study’s design, were collected using a mobile 3DO scanning application (MeThreeSixty, SizeStream LLC, Cary, NC, United States) with the front facing camera of an iPhone 14 Pro (Apple, Cupertino, CA, United States). Importantly, circumference estimates produced by this application have demonstrated excellent test-retest reliability and have shown to agree with traditional tape measurement.^24^ The following 3DO-derived circumferences, as titled and defined by the application manufacturer, were utilized based on their alignment with our protocols, as well as the body circumference measurement guidelines of the USAF: “CollarCircumference,” defined as the contour circumference of the neck column taken at the larynx; “StomachMaxCircumference,” defined as the horizonal circumference taken at the maximum circumference below the bust and above the waist; and “SeatCircumference” defined as the horizontal circumference at the most prominent rear point between the WC height and crotch height.

The procedures for 3DO scanning have been described in detail elsewhere.^24^^,^^25^ Briefly, cadets were required to wear tight-fitting athletic clothing (females: sports-bra/compression shorts; males: compression shorts only) and to tie their hair up, if necessary. For testing, the smartphone was secured to a stationary tripod at a standardized height and distance, then automated verbal prompts instructed cadets to stand in two distinct poses: a front facing pose with feet spread and hands laterally raised to the side, and a side facing pose with feet together and hands against their lateral thigh. A single image was collected during each pose and used to estimate BF%, as well as the aforementioned circumferences, using the application’s proprietary algorithms.

### AFROTC Anthropometric Equations

NC, WC, and HC were collected using a spring-loaded flexible aluminum tape measurement by a trained investigator, and the reliability of our measurement protocol has been presented in prior studies.^24^ NC was collected at an oblique angle positioned just above the Adam’s apple. WC was collected at the abdominal midpoint, defined as the midpoint between the iliac crest and lowest rib.^26^ HC was collected at the widest lateral portion of the hips. These circumferences, along with height, were subsequently used to estimate BF% using the equations employed by the USAF body composition measurement guidelines:^17^


Female BF%=163.205×(log⁡Waist+Hip-Neck)-97.684×(log⁡Height)-78.387

Male BF%=86.01×(log⁡Waist-Neck)-70.041×(log⁡Height)+36.76

where all circumferences are measured in inches. Circumferences from tape measure, along with those obtained using 3DO were used to produce two estimates: (1) AFROTC BF% equations based on tape measurements (AFROTC_STANDARD_) and (2) 3DO (AFROTC_3DO_).

### Smartwatch and Multifrequency Bioelectrical Impedance Analysis

BF% from SWBIA was assessed using a smartwatch with single-frequency, tetra-polar, hand-to-hand electrode placement (Galaxy Watch 6, Samsung, Suwon, South Korea), which has been described in detail elsewhere.^21^ The SWBIA was placed on the participants left wrist after ≥5-minutes of sitting.^22^ Then, participants were instructed to raise their hands in front of their body and place the fingers of the right hand on the SWBIA so that their middle and ring finger were in contact with the electrodes on the watch’s frame with their arms lifted to resemble a “circle” or “circuit”. Participants remained in this position for ∼15-seconds while tests were completed.

### Near-Infrared Reactance Spectroscopy

A portable time-resolved/regional NIRS device (Fitto, Olive Healthcare) was used to measure BF%, and the procedures for this assessment have been described in detail in prior investigations.^16^^,^^27^ Before scanning, user information was uploaded into a mobile application, which was then synced to the device itself. Then, automated visual prompts on the application directed the tester to scan specific body segments by placing the device flat against the participants: abdomen at 2.54 cm above the navel; midpoint of the right and left biceps brachii; and midpoint of the lower one-third of the right and left thigh. Using proprietary algorithms trained by DXA, the device uses the estimates obtained from each body region to estimate total BF%.

### Statistical Analyses

To assess cross-sectional validity, constant error was calculated as the mean difference between the BF% estimate from each device minus the criterion (DXA). Cross-sectional performance was assessed using paired *t*-tests, equivalence testing with ±2% equivalence regions,^11^ root mean squared error, standard error of the estimate, concordance correlation coefficients, coefficients of determination (R^2^), and both Deming regression and Bland-Altman analyses. Agreement with the line-of-identity via Deming regression was confirmed if the 95% confidence intervals for the intercept and slope included 0 and 1, respectively. Bland-Altman and linear regression techniques were used to determine the 95% limits of agreement and proportional bias. Longitudinal validity was assessed using the same statistical approach but compared ΔBF% over time between each device and DXA. Because of the small observed ΔBF% via DXA (−0.85%), longitudinal equivalence regions were defined as ±1% ΔBF%.^12^

Given that the primary application of body composition methods in AFROTC settings is to determine whether cadets meet established body composition standards, we evaluated each method’s ability to correctly distinguish between cadets meeting or not meeting the lower and upper body composition standards relative to DXA using binomial logistic regression. The true positive (meeting the body composition standards) and true negative proportions from each method were compared to those determined by DXA using the receiver operating characteristic area under the curve (AUC), sensitivity, specificity, and overall accuracy. Classification performance for each device was considered acceptable if AUC ≥0.70 and the summed sensitivity and specificity (SSS) was ≥1.50.^28-30^ Moreover, changes in these classifications over time were assessed using McNemar tests with corrections for continuity.^31^^,^^32^

A ranking system reported in prior studies^12^ was adapted and used to more effectively interpret the collective performance of each device across several domains. Cross-sectional and longitudinal validity at the group-level were ranked using root mean squared error, whereas individual-level validity was ranked using 95% limits of agreement. Rankings for clinical applicability were based on the SSS for each device. Devices were ranked from best (lowest score) to worst (highest score) for each domain, where smaller scores reflected better performance, and ties represented equal performance for the respective domain. Global performance scores, calculated as the sum of the 5 domains, were used to represent the final performance ranking of each device (1^st^-6^th^).^12^

## RESULTS

### Cross-Sectional Validity

The results for the cross-sectional validity of each method are depicted in Table 1 and Figure 1. Overall, 82.5% (*n* = 47) of cadets using SWBIA, 75.4% (*n* = 39) using NIRS, 64.9% (*n* = 37) using BIS, 56.1% (*n* = 32) using both AFROTC_3DO_ and 3DO (*n* = 32), and 38.6% (*n* = 22) using AFROTC_STANDARD_ produced BF% estimates within ±5% of those generated by DXA.

**Figure 1.**
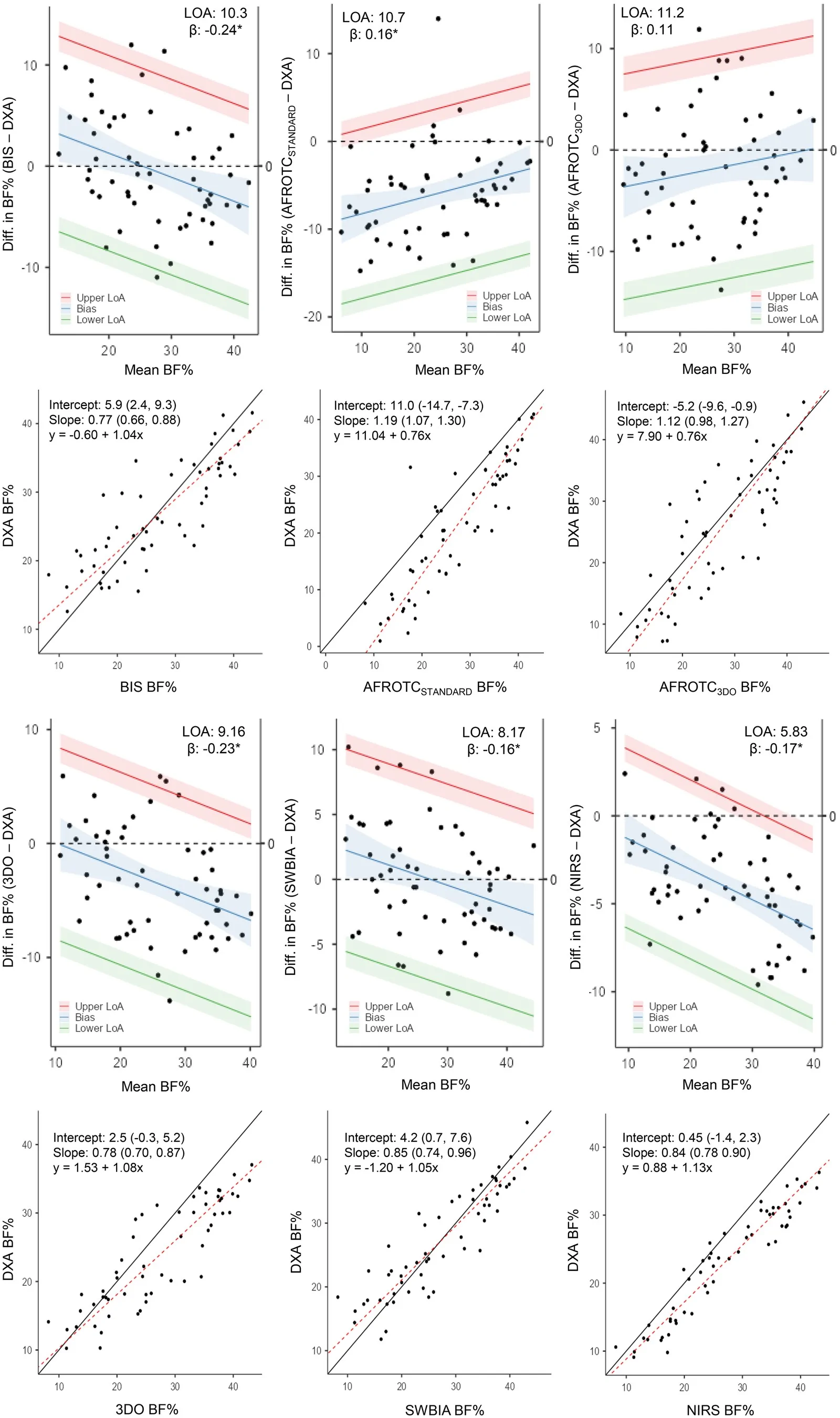
Bland-Altman And Deming regression plots demonstrating the cross-sectional agreement between each field method and DXA (*n* = 57). Bland-Altman plots are displayed in the top and third rows, while Deming regression plots are presented in the second and fourth rows. For the Bland-Altman plots, the upper and lower dashed lines and their associated shaded areas represent the 95% limits of agreement and their 95% confidence intervals, the middle-dashed line represents the mean difference in BF% between the method in question and DXA, and the solid blue line and its corresponding shaded area represents the regression line and its 95% CI, respectively. For the Deming regression plots, the solid black line represent the line of identity, and the red dashed line represents the regression. Deming regression results are presented as the intercept or slope (95% CI). β: proportional bias coefficient; LOA: 95% limits of agreement; BF%: body fat percentage; BIS: bioelectrical impedance analysis; DXA: dual-energy X-ray absorptiometry; AFROTC: U.S. Air Force Reserve Officers’ Training Corps; AFROTC_STANDARD_: AFROTC circumference-based method using manual circumference measurements; 3DO: smartphone-based three-dimensional optical scanning; AFROTC_3DO_: AFROTC circumference-based method with 3DO circumferences substituted for manual measurements; SWBIA: smartwatch-based bioelectrical impedance analysis; NIRS: near-infrared reactance spectroscopy. * statistically significant at *P* < .050.

Constant errors (−5.96% to −0.02%) were largest for AFROTC_STANDARD_ and smallest for SWBIA. BF% estimates from both BIS (*P* = .579) and SWBIA (*P* = .975) demonstrated equivalence with DXA and were not significantly different. All other methods significantly underestimated BF% (all *P* ≤ .018) and failed to demonstrate equivalence. Root mean squared errors (4.13-7.84) and standard errors of the estimate (1.44-3.64) were largest for AFROTC_STANDARD_, and smallest for SWBIA and NIRS, respectively, although 95% limits of agreement (±5.83-11.2) were largest for AFROTC_3DO_ and smallest for NIRS. R^2^ (0.70-0.91) and concordance correlation coefficients (0.75-0.89) were large, with the lowest produced by BIS and AFROTC_STANDARD_, respectively, and the highest from NIRS and SWBIA, respectively. Slopes of the regression line within Bland-Altman plots (β: −0.24-0.11) revealed that all methods significantly underestimated BF% at higher degrees of adiposity (*P* ≤ .014) with the exception of AFROTC_STANDARD_, which revealed significant overestimation (*P* = .019), and AFROTC_3DO_, which did not reveal significant proportional bias (*P* = .180). Deming regression revealed agreement with the slope of the line-of-identity for AFROTC_3DO_, and agreement with the intercept for both 3DO and NIRS. However, no device demonstrated agreement with both the slope and intercept simultaneously.

Results of the logistic regression models evaluating the ability of each method to correctly distinguish between cadets meeting or not meeting the lower and upper body composition standards relative to DXA are presented in Table S1. For BMI, 57.9% (*n* = 33) and 73.7% (*n* = 42) of cadets met lower and upper BMI cutoffs, respectively, whereas 33.3% (*n* = 19) and 63.2% (*n* = 36) of cadets met the lower and upper BF% cutoffs using DXA, respectively. For the lower cutoff, chi-square tests (χ^2^: 6.27-45.0) for overall model performance were significant for all methods (*P* ≤ .026), and diagnostic accuracy ranged from 71.9% to 89.5%. All methods with the exception of BIS (SSS: 1.31; AUC: 0.66) revealed SSS and AUCs >1.50 and 0.70, respectively. For the upper cutoff, chi-square tests (χ^2^: 12.7-25.5) remained significant for all methods (*P* ≤ .002), and diagnostic accuracy ranged from 75.4% to 84.2%. Only BIS, AFROTC_3DO_, and SWBIA revealed SSS and AUCs >1.50 and 0.70, respectively.

### Longitudinal Validity

The results for the longitudinal validity of each method are presented in Table 1 and Figure 2. Of those completing both baseline and follow-up testing (*n* = 50), 70% (*n* = 35) of cadets using 3DO, 64% (both *n* = 32) using both AFROTC equations, 62% (*n* = 31) using NIRS, 54% (*n* = 27) using SWBIA, and 30% (*n* = 15) using BIS produced ΔBF% estimates within ±2% of the ΔBF% measured by DXA. There were no significant changes in the distribution of participants across the lower or upper BMI or BF% cutoff groups (all *P* ≥ .221).

**Figure 2.**
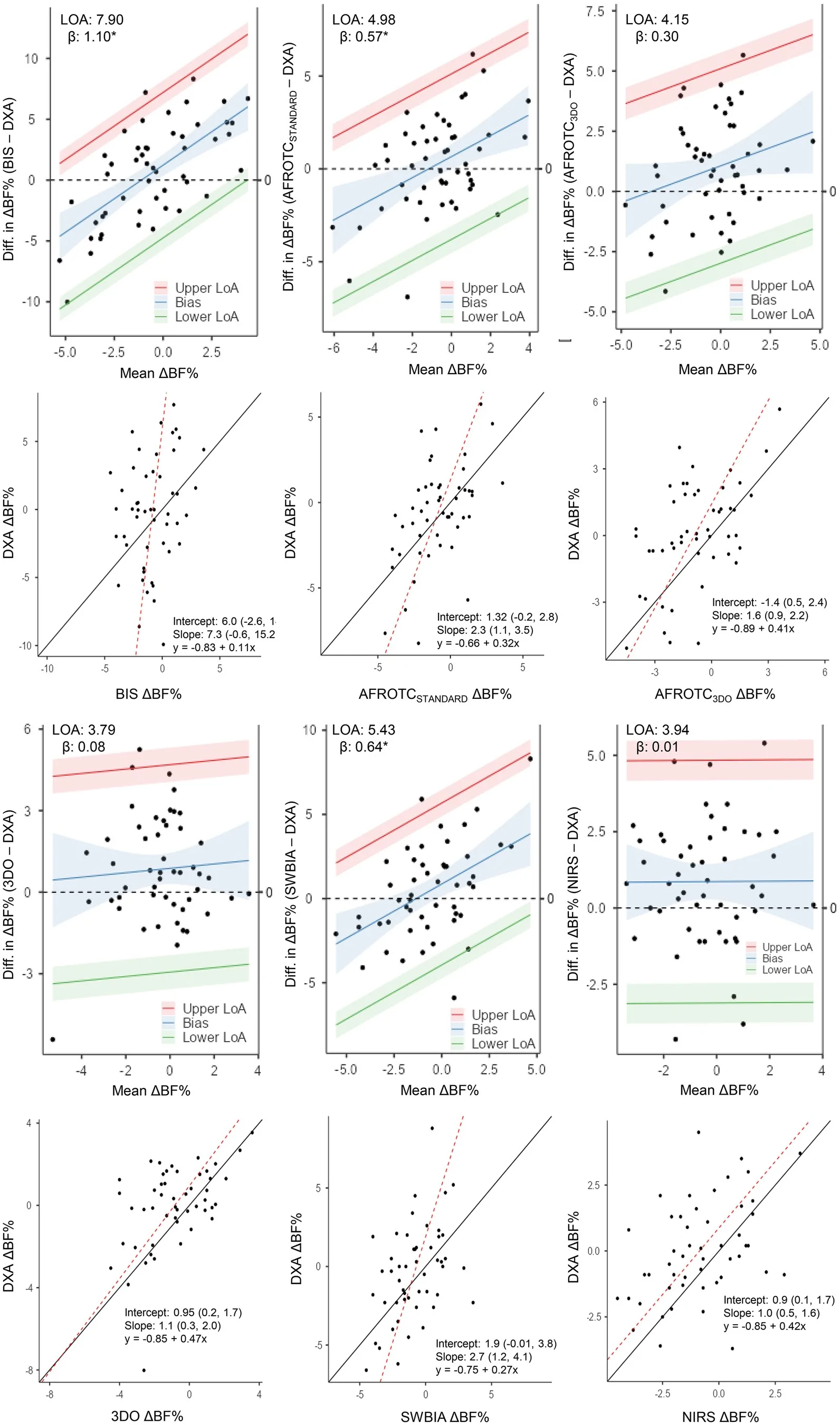
Bland-Altman And Deming regression plots demonstrating the longitudinal agreement between each field-method and DXA (*n* = 50). Bland-Altman plots are displayed in the top and third rows, although Deming regression plots are presented in the second and fourth rows. For the Bland-Altman plots, the upper and lower dashed lines and their associated shaded areas represent the 95% limits of agreement and their 95% confidence intervals, the middle-dashed line represents the mean difference in BF% change between the method in question and DXA, and the solid blue line and its corresponding shaded area represents the regression line and its 95% CI, respectively. For the Deming regression plots, the solid black line represents the line of identity, and the red dashed line represents the regression. Deming regression results are presented as the intercept or slope (95% CI). β: proportional bias coefficient; LOA: 95% limits of agreement; BF%: body fat percentage; BIS: bioelectrical impedance analysis; DXA: dual-energy X-ray absorptiometry; AFROTC: U.S. Air Force Reserve Officer Training Corps; AFROTC_STANDARD_: AFROTC circumferences-based method using manual circumference measurements; 3DO: smartphone-based three-dimensional optical scanning; AFROTC_3DO_: AFROTC circumferences-based method with 3DO circumferences substituted for manual measurements; SWBIA: smartwatch-based bioelectrical impedance analysis; NIRS: near-infrared reactance spectroscopy; Δ: change pre and post training program. * statistically significant at *P* < .050.

Constant errors for the ΔBF% between each device and DXA (0.25%-0.95%) were largest for AFROTC_3DO_ and smallest for AFROTC_STANDARD_. ΔBF% from BIS, SWBIA, and AFROTC_STANDARD_ (*P* ≥ .246) were not significantly different from DXA; however, only AFROTC_STANDARD_ demonstrated equivalence. All other methods significantly underestimated ΔBF% relative to DXA (*P* ≤ .004) and failed to demonstrate equivalence. Root mean squared errors (2.09-4.04), standard errors of the estimate (1.82-3.92), and 95% limits of agreement (±3.79-7.90) were largest for BIS and smallest for 3DO. R^2^ (0.06-0.27) and concordance correlation coefficients (0.18-0.46) were small-to-moderate, with the lowest produced from BIS and the highest produced by both AFROTC equations and 3DO. Slopes of the regression line within Bland-Altman plots (β: 0.01-1.10) revealed that BIS, AFROTC_STANDARD_, and SWBIA significantly underestimated ΔBF% at higher degrees of change (all *P* < .001), which was not observed for the remaining devices (*P* ≤ .064). Deming regression revealed agreement with the slope of the line-of-identity for all but SWBIA and AFROTC_STANDARD_, and agreement with the intercept for all but 3DO, NIRS, and AFROTC_3DO_. Only BIS demonstrated agreement with both the slope and intercept simultaneously.

### Overall Device Performance

Table 2 presents the rankings of each device based on cross-sectional and longitudinal validity at both group and individual levels, along with their clinical application scores. Overall, NIRS ranked first, SWBIA second, and 3DO third. NIRS and SWBIA tied for the highest cross-sectional validity, although 3DO ranked highest for longitudinal validity. For clinical applicability, SWBIA ranked first, followed by NIRS.

**Table 2.** Global Performance of Each Device Ranked by Clinical Applicability and Cross-Sectional and Longitudinal Validity Scores

					Group-level validity		Individual-level validity		
	Global rank	Global sum	Cross-sectional rank	Longitudinal rank	Cross-sectional	Longitudinal	Cross-sectional	Longitudinal	Clinical applicability Ranking
NIRS BF%	1	9	1	2	2	2	1	2	2
Smartwatch BIA BF%	2	14	1	5	1	5	2	5	1
Smartphone 3DO BF%	3	15	2	1	4	1	3	1	6
AFROTC_3DO_ BF%	4	20	3	3	4	3	6	3	3
AFROTC_STANDARD_ BF%	5	23	3	4	6	4	5	4	4
BIS BF%	6	24	2	6	3	6	4	6	5

Devices are ranked from best (lowest score) to worst (highest score) for each column and ties represent equal performance for the respective column.

Group-level validity is ranked using root mean squared error, individual-level validity is ranked using 95% limits of agreement and clinical applicability is ranked using summed sensitivity and specificity.

Global sums are calculated as the sum of the clinical applicability and the group and individual cross-sectional and longitudinal validity rankings.

Abbreviations: 3DO, smartphone-based three-dimensional optical scanning; 3DO, smartphone-based three-dimensional optical scanning; AFROTC, U.S. Air Force Reserve Officer Training Corps; AFROTC_3DO_, AFROTC circumferences-based method with 3DO circumferences substituted for manual measurements; AFROTC_STANDARD_, AFROTC circumferences-based method using manual circumference measurements; BF%, body fat percentage; BIA: bioelectrical impedance analysis; BIS, bioelectrical impedance analysis; NIRS, near-infrared reactance spectroscopy.

## DISCUSSION

Although most cadets met the USAF BMI standards, a substantial proportion did not meet the DXA-derived USAF BF% standards, reinforcing that BMI alone fails to differentiate between physically prepared cadets and those with excessive adiposity. This commonly reported pattern^6^ highlights the flaws of BMI as a physical readiness benchmark, supporting the importance of evaluating body composition beyond weight-per-height. Overall, our field-based tools demonstrated the most valid and practical alternatives to DXA, making them a viable option for body composition monitoring within ROTC and USAF settings.

Our finding of high false compliance has important consequences for readiness assessments. BMI not only penalizes muscular cadets but also fails to identify those with insufficient lean mass, potentially resulting in unfair disqualifications and increased injury risk,^7^ before considering the safety implications for the individual and their teammates in mission-critical environments. In contrast, estimated BF% offers a more accurate and functional indicator of performance potential and injury susceptibility, emphasizing the need to move beyond BMI and, based on our findings of poor performance, circumference-based BF% standards in military contexts. For cross-sectional performance, BF% estimates produced by NIRS and SWBIA most closely aligned with DXA, each demonstrating excellent accuracy and the ability to correctly classify cadets based on USAF BF% standards.

SWBIA is particularly promising given its existing integration within the wearable technologies already used by cadets, enabling seamless and continuous monitoring without added burden or interference. In fact, the expanding role of wearable devices in military health monitoring is highlighted in recent USAF interest in this technology, where the U.S. Space Force has recently initiated testing smartwatches within its fitness and readiness programs.^33^ Conversely, NIRS offers a low-cost, portable alternative that can provide estimates for large detachments with minimal training. Although 3DO was slightly less accurate (based on our global performance scores) cross-sectionally than SWBIA and NIRS, its accessibility makes it highly practical for large-scale implementation. Manual circumference-based equations, however, produced the largest, potentially disqualifying (<50% of estimates ±5% of reference) errors, reinforcing long-standing concerns regarding their reliability.^31^ Overall, these digital body composition technologies represent scalable, field-ready solutions for improving the accuracy and accessibility of body composition assessments in military and other tactical populations.

Despite small overall shifts in BF%, 3DO demonstrated the highest accuracy (based on our global performance scores) in tracking longitudinal changes relative to DXA. Although NIRS and SWBIA were slightly less sensitive to detecting longitudinal changes, their consistent performance across time exhibits their reliability during repeated field assessments. Collectively, these findings highlight the value of automated technologies for minimizing human error, improving standardization, and supporting both baseline classification and ongoing monitoring in AFROTC cadets.

## CONCLUSIONS

Reliance on BMI or circumference-based BF% estimates alone remain problematic for assessing cadet readiness. Instead, NIRS, SWBIA, and 3DO provide accurate, portable alternatives for assessing BF% in AFROTC cadets that can be deployed at scale with minimal burden. Moreover, these automated technologies enable cadre to obtain real-time, standardized data that can more accurately assess body composition, identify cadet potential, and evaluate PT program effectiveness. Adopting accessible, user-friendly technologies could improve fairness and accuracy in military fitness assessments and strengthen readiness standards through better reflection of true physical capabilities, resolving current limitations of traditional anthropometric methods. Future research should validate these methods in larger cohorts (single-cohort studies may not depict the spectrum of body morphologies across other U.S. detachments) undergoing varied training programs across different operational settings.

## Supplementary Material

usag107_Supplementary_Data

## Data Availability

The datasets used and/or analyzed during the current study are available from the corresponding author on reasonable request.

## References

[1] Mackey CS , DeFreitas JM. A longitudinal analysis of the U.S. Air force reserve officers’ training corps physical fitness assessment. Mil Med Res. 2019;6:30. 10.1186/s40779-019-0219-4 PMC6755694 31543076

[2] Naylor TR , Kiss JE, Fullenkamp AM, Garon DK. Comparing body composition methods for air force reserve officers’ training corps cadets. Mil Med. 2024;189:e274–e278, 10.1093/milmed/usad311 37646781

[3] Foulis SA , Friedl KE, Spiering BA, et al Body composition changes during 8 weeks of military training are not accurately captured by circumference-based assessments. Front Physiol. 2023;14:1183836. 10.3389/fphys.2023.1183836 PMC10282178 37351259

[4] Latour AW , Peterson DD, Riner DD. Comparing alternate percent body fat estimation techniques for United States navy body composition assessment. Int J Kinesiol High Educ. 2019;3:93–105. 10.1080/24711616.2019.1584547

[5] Florez CM , Rodriguez C, Siedler MR, Tinoco E, Tinsley GM. Body composition estimation from mobile phone three-dimensional imaging: evaluation of the USA army one-site method. Br J Nutr. 2024;132:1–9. 10.1017/S0007114524002216 PMC11617106 39411840

[6] Meyer S , Cole R. Army body composition program study results concerning: enrollees are more over fat than expected. Mil Med. 2019;184:400–8. 10.1093/milmed/usy302 30901401

[7] Knapik JJ , Farina EK, Steelman RA, Trone DW, Lieberman HR. The medical burden of obesity and overweight in the US military: association of BMI with clinically diagnosed medical conditions in United States military service members. J Nutr. 2023;153:2951–67. 10.1016/j.tjnut.2023.08.023 37619919

[8] Reyes-Guzman CM , Bray RM, Forman-Hoffman VL, Williams J. Overweight and obesity trends among active duty military personnel: a 13-year perspective. Am J Prev Med. 2015;48:145–53. 10.1016/j.amepre.2014.08.033 25442226

[9] Johnson QR , Mackey CS, Muddle TD, Smith DB, DeFreitas JM. Body composition comparison of upper- and underclass reserve officers’ training corps cadets. Aerosp Med Hum Perform. 2019;90:813–8. 10.3357/AMHP.5355.2019 31426898

[10] Moon JR , Tobkin SE, Smith AE, et al Percent body fat estimations in college men using field and laboratory methods: a three-compartment model approach. Dyn Med. 2008;7:7. 10.1186/1476-5918-7-7 PMC2365941 18426582

[11] Tinsley GM , Harty PS, Stratton MT, Smith RW, Rodriguez C, Siedler MR. Tracking changes in body composition: comparison of methods and influence of pre-assessment standardisation. Br J Nutr. 2022;127:1656–74. 10.1017/S0007114521002579 34325758

[12] Siedler MR , Rodriguez C, Stratton MT, et al Assessing the reliability and cross-sectional and longitudinal validity of fifteen bioelectrical impedance analysis devices. Br J Nutr. 2023;130:827–40. 10.1017/S0007114522003749 PMC10404482 36404739

[13] Feeney KA , Melton BF, Ryan GA, Bland HW, Butler CR. The association between body composition and performance on military fitness tests. J Strength Cond Res. 2025;39:94–8. 10.1519/JSC.0000000000004952 39652732

[14] McClung HL , Bartlett PM, Spiering BA, et al Science behind policy: implementing a modern circumference-based body fat equation with a physical fitness threshold is associated with lower musculoskeletal injury risk. Int J Obes (Lond). 2025;49:723–30. 10.1038/s41366-024-01701-5 PMC11999870 40016560

[15] Haynes H , Tinsley GM, Swafford SH, et al Mobile anthropometry in division i baseball athletes: evaluation of an existing application and the development of new equations. J Strength Cond Res. Published online 2025;39:447–56. 10.1519/JSC.0000000000005039 39714226

[16] Brandner CF , Compton AT, Swafford SH, et al Total and appendicular body composition comparisons between near-infrared reactance spectroscopy and dual energy X-ray absorptiometry. Clin Physiol Funct Imaging. 2025;45:e12914. 10.1111/cpf.12914 39513679

[17] Department of Defense. DoD Instruction 1308.03 DOD Physical Fitness/Body Composition Program. Department of Defense; 2022. Updated June 25, 2025. Accessed October 8, 2025. https://www.esd.whs.mil/Portals/54/Documents/DD/issuances/dodi/130803p.pdf

[18] Graybeal AJ , Swafford SH, Compton AT, Renna ME, Thorsen T, Stavres J. Predicting bone mineral content from smartphone digital anthropometrics: evaluation of an existing application and the development of new prediction models. J Clin Densitom. 2025;28:101537. 10.1016/j.jocd.2024.101537 PMC11781973 39509826

[19] Moço AV , Matias CN, Santos DA, et al Usefulness of reflection scanning in determining whole-body composition in broadly built individuals using dual-energy X-ray absorptiometry. J Clin Densitom. 2019;22:429–36. 10.1016/j.jocd.2018.03.007 29681438

[20] Tinsley GM , Moore ML, Graybeal AJ. Precision of dual-energy X-ray absorptiometry reflection scans in muscular athletes. J Clin Densitom. 2020;23:647–55. 10.1016/j.jocd.2018.09.005 30327241

[21] Brandner CF , Tinsley GM, Graybeal AJ. Smartwatch-based bioimpedance analysis for body composition estimation: precision and agreement with a 4-compartment model. Appl Physiol Nutr Metab. 2023;48:172–82. 10.1139/apnm-2022-0301 36462216

[22] Vallecillo-Bustos A , Compton AT, Swafford SH, et al The effect of postural orientation on body composition and total body water estimates produced by smartwatch bioelectrical impedance analysis: an intra- and inter-device evaluation. J Electr Bioimpedance. 2024;15:89–98. 10.2478/joeb-2024-0010 PMC11299788 39105154

[23] Moon JR , Tobkin SE, Roberts MD, et al Total body water estimations in healthy men and women using bioimpedance spectroscopy: a deuterium oxide comparison. Nutr Metab (Lond). 2008;5:7. 10.1186/1743-7075-5-7 PMC2323003 18353180

[24] Graybeal AJ , Brandner CF, Tinsley GM. Evaluation of automated anthropometrics produced by smartphone-based machine learning: a comparison with traditional anthropometric assessments. Br J Nutr. 2023;130:1077–87. 10.1017/S0007114523000090 PMC10442791 36632007

[25] Graybeal AJ , Brandner CF, Tinsley GM. Visual body composition assessment methods: a 4-compartment model comparison of smartphone-based artificial intelligence for body composition estimation in healthy adults. Clin Nutr. 2022;41:2464–72. 10.1016/j.clnu.2022.09.014 36215866

[26] Graybeal AJ , Compton AT, Swafford SH, et al Measurements of abdominal obesity are associated with metabolic syndrome severity independent of hypertensive phenotype in white but not black young adults. J Racial Ethn Health Disparities. 2025;12:2299–311. 10.1007/s40615-024-02051-8 38902464

[27] Graybeal AJ , Brandner CF, Wise HL, et al Near-infrared reactance spectroscopy-derived visceral adipose tissue for the assessment of metabolic syndrome in a multi-ethnic sample of young adults. Am J Hum Biol. 2024;36:e24141. 10.1002/ajhb.24141 39034709

[28] Graybeal AJ , Compton AT, Swafford SH, et al A predictive model for body water and fluid balance using 3D smartphone anthropometry. Front Physiol. 2025;16:1577049. 10.3389/fphys.2025.1577049 PMC12230031 40626043

[29] Mandrekar JN. Receiver operating characteristic curve in diagnostic test assessment. J Thorac Oncol. 2010;5:1315–6. 10.1097/JTO.0b013e3181ec173d 20736804

[30] Power M , Fell G, Wright M. Principles for high-quality, high-value testing. Evid Based Med. 2013;18:5–10. 10.1136/eb-2012-100645 PMC3585491 22740357

[31] Graybeal AJ , Brandner CF, Compton AT, et al Differences in metabolic syndrome severity and prevalence across nine waist circumference measurements collected from smartphone digital anthropometrics. Clin Nutr ESPEN. 2024;64:390–9. 10.1016/j.clnesp.2024.10.158 39486478

[32] Graybeal AJ , Kreutzer A, Willis JL, Moss K, Braun-Trocchio R, Shah M. Age drives the differences in dietary supplement use in endurance athletes: a cross-sectional analysis of cyclists, runners, and triathletes. J Diet Suppl. 2023;20:602–20. 10.1080/19390211.2022.2056670 35380079

[33] Air Force Research Laboratory. AFRL leads USSF Continuous Fitness Assessment study. *Win the Future*. June 22, 2023. Accessed October 24, 2025. https://www.afrl.af.mil/News/Article-Display/Article/3433012/afrl-leads-ussf-continuous-fitness-assessment-study/

