# Supplementary material for: Cross-Sectional and Longitudinal Validity of Automated Body Composition Methods in U.S. Air Force Reserve Officers’ Training Corps Cadets Throughout a 12-Week Physical Training Program: The Supporting Tactical Athlete Readiness Program
Source: Mil Med. 2026 Apr 3;191(11-12):e2413–24. doi: 10.1093/milmed/usag107 (PMC13730446; doi:10.1093/milmed/usag107)
Supplement: Supplementary file 1 [file usag107_supplementary_data.pdf]

Supplemental Table 1. Accuracy of Field-based Body Composition Methods in Correctly Classifying DXA-derived Body Fat Percentage According to AFROTC Standards

	DXA – Lower Cutoff [a]						DXA – Upper Cutoff [b]					
Cross-sectional	χ^2	Sensitivity	Specificity	SSS	Accuracy	AUC	χ^2	Sensitivity	Specificity	SSS	Accuracy	AUC
BIS	6.27*	0.47	0.84	1.31	71.9%	0.66	25.5*	0.94	0.67	1.61	84.2%	0.81
$AFROTC_{STANDARD}$	23.6*	0.95	0.68	1.63	77.2%	0.82	12.7*	0.97	0.38	1.35	75.4%	0.68
$AFROTC_{3DO}$	18.8*	0.74	0.84	1.58	80.7%	0.79	22.1*	0.92	0.67	1.59	82.5%	0.79
Smartphone 3DO	15.0*	0.74	0.79	1.53	77.2%	0.76	15.7*	1.00	0.33	1.33	75.4%	0.67
SWBIA	30.6*	0.68	0.97	1.65	87.7%	0.83	25.5*	0.94	0.67	1.61	84.2%	0.81
NIRS	45.0*	1.00	0.84	1.84	89.5%	0.92	18.3*	1.00	0.38	1.38	77.2%	0.69

[a] lower cutoff defined BF% < 18% for males and < 26% for females

[b] upper cutoff defined as BF% < 26% for males and < 36% for females

* statistically significant overall model test indicating good model performance at $p < 0.050$.

DXA: dual-energy X-ray absorptiometry; AFROTC: U.S. Air Force Reserve Officer Training Corps; SSS: summed sensitivity and specificity; receiver operating characteristic area under the curve; BIS: bioelectrical impedance analysis; $AFROTC_{STANDARD}$: AFROTC circumferences-based method using manual circumference measurements; 3DO: smartphone-based three-dimensional optical scanning; $AFROTC_{3DO}$: AFROTC circumferences-based method with 3DO circumferences substituted for manual measurements; SWBIA: smartwatch bioelectrical impedance analysis; NIRS: near-infrared reactance spectroscopy.
